# Supplementary material for: Spatiotemporal patterns of anemia among lactating mothers in Ethiopia using data from Ethiopian Demographic and Health Surveys (2005, 2011 and 2016)
Source: PLoS One. 2020 Aug 6;15(8):e0237147. doi: 10.1371/journal.pone.0237147 (PMC7410320; doi:10.1371/journal.pone.0237147)
Supplement: Attachment — Submitted filename: Reviewer Report.docx [file pone.0237147.s001.docx]

**Reviewer’s Report**

Spatial patterns of anemia among lactating mothers in Ethiopia: Data from Ethiopian Demographic and Health Surveys (2005, 2011 and 2016)

Anemia among human population is a major public health problem that affects populations in both rich and poor countries. Its primary cause is iron deficiency (IDA) and other various biological and non-biological factors. Anaemia is a common health problem in Ethiopia also where the latest DHS survey found around 24 percent women of reproductive age (WRA) were anaemic, ranges from a low of around only 16 percent to a high of close to 60 percent in different parts of Ethiopia. In the context, the spatial exploration of anaemia especially among lactating women is also an important issue and the authors have tried to study the concentration and clustering of one of the major health problems but the authors have missed somewhere and needs further improvement in the manuscripts. The constructive comments and suggestions are as follows-

1. Introduction section has started in right way with definition and cause of anaemia with most related to biological factors but missing non-biological factors. Next, anaemia as a public health problem has discuses in brief flowed by global scenario of Anaemia. Then adverse effect of maternal anaemia has been discussed before moving on scenario of anaemia in Ethiopia. Introduction section ends with reason for selecting lactating women for the study purpose.
2. Here, some important aspects found missing. Like, previous studies pertain to anaemia among WRA in Ethiopia, evidences and studies related to spatial pattern of anaemia among WRA in the study country. Reason for considering lactating women for study needs to be strengthened. Authors are also advised to do review of literature extensively as many relevant studies is available on anaemia among WRA in Ethiopia conducted on same data source at different point of time. Some studies are very recent with latest round of country DHS data.
3. Data source, sampling design and setting needs to be rewrite in appropriate manner. Whereas measurements should be different sections and can be written accordingly. It would have been better if researchers would have provided region wise sample over the three study period.
4. As the author mentioned “The haemoglobin level was measured for those eligible mothers after having consent and it was adjusted for altitude”. This needs to be clarified as DHS data for the country provide measured haemoglobin level and the adjusted haemoglobin for altitude. So, which one indicator authors have used in the study. The cut off used in this study has not been mentioned in the methodology section.
5. Spatial analysis section starts with autocorrelation analysis to spatial scan statistical analysis. Here, the concern is that in many section, authors have provided only the theoretical notions of such the method but missed to elaborate in accordance and need of the undertaken study. Like, how SAC has been executed in the study has found missing. Second, section on SAC mentioned somewhere “leads to rejection of null hypothesis and indicates the presence of spatial correlation”. Does the author have posed research question in same way?
6. Hot spot analysis (HSA) using Gettis-OrdGi* statistics is a appropriate way to check the variation in spatiality across the study areas but missed to the process undertaken in the study. It would be better if authors had been reviewed available study for spatial analysis of anaemia in Ethiopia by Kelemu T.K, et (2019) study using latest wave of Ethiopia DHS data. So, author is advised to go through the paper and restructure the section appropriately.
7. Overall methodology section needs to tightened and strengthened.
8. Results are very poorly interpreted, seems researchers are in hurry. So, results sections had to be written in well manner. Figures 3 can be interpreted region wise and changes over period would be better idea. Interpretation in figure heading is not the academic practices, so author is suggested to follow the rigour and pattern/outline to prepare the manuscript.
9. It would have been better to elaborate the section on emerging hotspot areas based on the data available for the areas before doing interpolation analysis to detect the primary and secondary clusters.
10. In spatial scan analysis, authors have tires to provide glimpse of significant clusters separately for each survey period but it they can provide reason wise prominent clusters, it would be better for planning and programme intervention at the regional level within Ethiopia.
11. Table 3 does not have catchy and reliable approach. It would have been better if the researchers would have been mentioned detailed with name or within each of the cluster with maximum and minimum concentration of anaemic lactating mothers. Second, researcher can match for enumeration areas which were found common during the period of 2005, 2011 and 2016, cluster wise. The format of table 3 provided in the text can be produced as supplementary table or appendix table.
12. Discussion section needs to rewrite as it is just findings of study only in very limited way. It is important to discuss the findings of the study with other previous and existing studies on the issues.
13. In the conclusion section authors have mentioned “Findings suggest that giving priority attentions would be important on water, and other nutrition-related interventions on the identified hotspot areas to prevent and control anaemia incidence among lactating mothers”. The concern is that from where these findings emerged as it is not found anywhere and anyway in the manuscript.
14. Maps and figures are also not readable as are very hazy. So authors are also advised to rework for figure and maps to make it clear before submitting it the journal.

Overall, the paper needs to revise with appropriate framework for the country undertaken for the policy and programmatic point of view.
