# Supplementary material for: Spatiotemporal patterns of anemia among lactating mothers in Ethiopia using data from Ethiopian Demographic and Health Surveys (2005, 2011 and 2016)
Source: PLoS One. 2020 Aug 6;15(8):e0237147. doi: 10.1371/journal.pone.0237147 (PMC7410320; doi:10.1371/journal.pone.0237147)
Supplement: Attachment — Submitted filename: response to reviewers.docx [file pone.0237147.s002.docx]

**Rebuttal letter	Date 1/27/2020**

**PONE-D-19-30322**

**Spatial Patterns of Anemia among Lactating Mothers in Ethiopia: Data from Ethiopian Demographic and Health Surveys (2005, 2011 and 2016)**

**Alemneh Mekuriaw Liyew**

**Plos One**

Dear all,

We would like to thank for these constructive, building and improvable comments on this manuscript that would improve substance and content of the manuscript. We considered each comments and clarification questions of editors and reviewers on the manuscript thoroughly. Our point-by-point responses for each comment and questions are described in detailed on the following pages. Further, the details of changes were shown by track changes in the supplementary document attached. The manuscript language was checked by language professionals and we follow journal guideline. I have attached recent comments in point by point response.

**Version**

**Comments from reviewer**

**Comment 1**: Introduction section has started in right way with definition and cause of anaemia with most related to biological factors but missing non-biological factors. Next, anaemia as a public health problem has discuses in brief flowed by global scenario of Anaemia. Then adverse effect of maternal anaemia has been discussed before moving on scenario of anaemia in Ethiopia. Introduction section ends with reason for selecting lactating women for the study purpose.

Response 1: Thanks in advance both editor and reviewer for your critical view to advance the quality of our manuscript. We improved accordingly.

Comment 2: “Here, some important aspects found missing. Like, previous studies pertain to anaemia among WRA in Ethiopia, evidences and studies related to spatial pattern of anaemia among WRA in the study country. Reason for considering lactating women for study needs to be strengthened. Authors are also advised to do review of literature extensively as many relevant studies is available on anaemia among WRA in Ethiopia conducted on same data source at different point of time. Some studies are very recent with latest round of country DHS data.”

Response 2: Thanks in advance both editor and reviewer for comments. First based on your constructive comment we have improved the justification for why we focused on lactating mothers accordingly. The introduction section Line 23-30 Page 1. Second, even though we considered some publications on anemia among reproductive age women ,our primary intention was focusing on the studies conducted on anemia among lactating mothers. However, your comment strongly convinced as. Therefore, we added the literatures on women of reproductive age like “ Kibret KT, Chojenta C, D’Arcy E, Loxton D. Spatial distribution and determinant factors of anaemia among women of reproductive age in Ethiopia: a multilevel and spatial analysis. BMJ open. 2019;9(4):e027276 and Ejigu BA, Wencheko E, Berhane K. Spatial pattern and determinants of anaemia in Ethiopia. PloS one. 2018;13(5” and others.

Comment 3: Data source, sampling design and setting needs to be rewrite in appropriate manner. Whereas measurements should be different sections and can be written accordingly. It would have been better if researchers would have provided region wise sample over the three study period.

Response 3: Thank you reviewer and editor for your constructive comment. We really appreciated the comment and totally converted sample to region wise approach since the further analysis was region based it is good to provide region based sampling information for the data set. **Method section Line 100, page 4.** In addition the “Data measurement” was provided in separate section as per your comment and data source, sampling design and setting were strengthened accordingly.

Comment 4: “As the author mentioned “The haemoglobin level was measured for those eligible mothers after having consent and it was adjusted for altitude”. This needs to be clarified as DHS data for the country provide measured haemoglobin level and the adjusted haemoglobin for altitude. So, which one indicator authors have used in the study. The cut off used in this study has not been mentioned in the methodology section.”

Response 4: Thanks reviewer and editor. This section was rewritten again and corrected as follows. “In Ethiopia, Data collection on anemia as key indicator in national surveys was started in 2005 survey and continued in the next two surveys (2011 and 2016). In all surveys , the hemoglobin level was measured for those eligible mothers after having consent and it was adjusted for altitude (18-20). Therefore, the current study was based on the altitude adjusted haemoglobin level which was already provided in the EDHS data. Finally, the women with hemoglobin level <120 g/L were considered as anemic. “**Method section Line 89-94, page 4**. This cut off point was used from WHO definition of anemia for non-pregnant women as it was indicated in background section.

Comment 5: Spatial analysis section starts with autocorrelation analysis to spatial scan statistical analysis. Here, the concern is that in many section, authors have provided only the theoretical notions of such the method but missed to elaborate in accordance and need of the undertaken study. Like, how SAC has been executed in the study has found missing. Second, section on SAC mentioned somewhere “leads to rejection of null hypothesis and indicates the presence of spatial correlation”. Does the author have posed research question in same way?

Response 5: Thanks reviewer and editor. We have rewritten this section under heading “Spatial analysis” just by avoiding the subsections. We paraphrased each section as a single paragraph in coherent way which shows the sequence of the spatial analysis methods conducted. We again indicated the purpose of conducting each analysis methods in relation with the theoretical notion of the methods. The second point here was the concept of the following sentence. “Leads to rejection of null hypothesis and indicates the presence of spatial correlation”. Does the author have posed research question in same way?” This sentence was used just to indicate that how the spatial autocorrelation works. When we conduct spatial autocorrelation analysis generally the **null hypothesis** was random distribution of the disease of interest over the study area. Therefore when we get significant global Moran’s index in spatial autocorrelation analysis result we reject the null hypothesis and deal with spatial clustering to detect further the local level clusters by using other analytic methods like hotspot analysis and spatial scan statistics. It was incorporated to indicate this general concept. However we have rephrased in the revised manuscript to avoid such confusion. **Method section Line 106-145 Page 6**

Comment 6: Hot spot analysis (HSA) using Gettis-OrdGi* statistics is a appropriate way to check the variation in spatiality across the study areas but missed to the process undertaken in the study. It would be better if authors had been reviewed available study for spatial analysis of anaemia in Ethiopia by Kelemu T.K, et (2019) study using latest wave of Ethiopia DHS data. So, author is advised to go through the paper and restructure the section appropriately.

Response 6: Thanks reviewer for your commitment just to improve our manuscript by searching such relevant studies. I hope we have benefited a lot to strengthen the manuscript. Therefore we have restructured the hotspot analysis and even other sections accordingly as I have described in response 5.

Comment 7: Overall methodology section needs to tightened and strengthened

Response 7: Thanks reviewer. Based on recommendation we go through the method section organized it as it was indicated in the track change document.

Comment 8: Results are very poorly interpreted, seems researchers are in hurry. So, results sections had to be written in well manner. Figures 3 can be interpreted region wise and changes over period would be better idea. Interpretation in figure heading is not the academic practices, so author is suggested to follow the rigour and pattern/outline to prepare the manuscript.

Response 8: Thank you reviewer for your valuable comment. Based on your comment the result section is rewritten again. We have avoided the interpretations in the figure heading and adjusted it according to the guideline in all figures. The source for the shape file for maps was also indicated at the figure heading.

Comment 9: It would have been better to elaborate the section on emerging hotspot areas based on the data available for the areas before doing interpolation analysis to detect the primary and secondary clusters.

Response 9: thanks reviewer. We provided the prominent risk areas based on the spatial scan statistics results and the spatial interpolation was conducted to detect the predicted risk areas for anemia in unsampled areas.

Comment 10: In spatial scan analysis, authors have tires to provide glimpse of significant clusters separately for each survey period but it they can provide reason wise prominent clusters, it would be better for planning and programme intervention at the regional level within Ethiopia.

Response 10: Thanks reviewer since the study involved the three surveys separately we were ended up with a number significant clusters. As per your comment currently we have focused on the most likely clusters only in each survey. As you justified in your comment, even the purpose of conducting spatial scan statistics than the hotspot analysis is to detect most prominent cluster for intervention especially in resource limited areas. Therefore, we welcomed your really constructive comment and acted accordingly.

Comment 11: Table 3 does not have catchy and reliable approach. It would have been better if the researchers would have been mentioned detailed with name or within each of the cluster with maximum and minimum concentration of anaemic lactating mothers. Second, researcher can match for enumeration areas which were found common during the period of 2005, 2011 and 2016, cluster wise. The format of table 3 provided in the text can be produced as supplementary table or appendix table

Response 11: thanks reviewer. Here again we appreciated your comment and reorganized the table by focusing on most likely clusters across each survey. However, we fail to provide the enumeration areas which were found common during three surveys. This was because the enumeration areas used in three periods were different in their number and type as it was indicated in the method section.

Comment 12: Discussion section needs to rewrite as it is just findings of study only in very limited way. It is important to discuss the findings of the study with other previous and existing studies on the issues.

Response 12: thanks reviewer as per your recommendation we reorganized the discussion in line with the adjustments made in result section.

Comment 13: In the conclusion section authors have mentioned “Findings suggest that giving priority attentions would be important on water, and other nutrition-related interventions on the identified hotspot areas to prevent and control anaemia incidence among lactating mothers”. The concern is that from where these findings emerged as it is not found anywhere and anyway in the manuscript.

Response 13: thank you reviewer for your wonderful comment. We have completely avoided this sentence and organized accordingly. **Line 289-298 page 12.**

Comment 14: Maps and figures are also not readable as are very hazy. So authors are also advised to rework for figure and maps to make it clear before submitting it the journal.

Response 14: thank you reviewer. We reworked all the figures and maps carefully. We hope all they are readable and clear in the revised manuscript.

Over all the comments were wonderful and we learn a lot from the comments.

**Version two**

**Issues raised by academic editor**

Thank you editor once again for critical consideration of our manuscript. Here is to declare that we have adjusted the following points based on your recommendation. We also reflected accordingly on the issues which require further clarification.

1. We have checked that our manuscript meets the plos one requirement

2. We have rephrased to avoid the text overlap with the previous publications.

3. We have included the potential limitations of the study in the discussion section.

4. Thank you editor for the constructive comments. Here we are interested to clarify the source of the figures. We kindly declare that all the figures and maps are our own works. The shape file for those maps was accessed from Ethiopian Central Statistical Agency and the geographic coordinates were accessed from measure DHS after being authorized and registered user. Therefore, we prepared all the maps used to show the spatial analysis in each survey by ArcGIS10.7 software. We indicated the source of the shape file at the right lower corner of each map and for more clarity we have also included the source of the shape file at the figure heading in all figures.

5. I the crospondng author included the ORCID in the revised submission.
