# Supplementary material for: Spatiotemporal patterns of anemia among lactating mothers in Ethiopia using data from Ethiopian Demographic and Health Surveys (2005, 2011 and 2016)
Source: PLoS One. 2020 Aug 6;15(8):e0237147. doi: 10.1371/journal.pone.0237147 (PMC7410320; doi:10.1371/journal.pone.0237147)
Supplement: Attachment — Submitted filename: Reviewer Comments08 April 2020.docx [file pone.0237147.s003.docx]

**Reviewer Comments**

**Spatial Patterns of Anemia among Lactating Mothers in Ethiopia: Data from Ethiopian Demographic and Health Surveys (2005, 2011 and 2016)**

**Although, the revised paper shows improvement than the previous one but, still there is need to work on it to improve the quality and strength of the paper to meet the criteria of the said journal. Important question is why the spatial hotspot is in those areas. Using data for three points of time to make it comparable also needs logically justification. Some other important comments are as follows-**

1. Keep the use of term consistent- like somewhere author used maternal, somewhere lactating and somewhere breastfeeding mothers.
2. Line no 50 are not well connected with line no 49.
3. Authors need to rewrite the data source and sampling section and data measurement section. As these two are overlapping.
4. Authors missed to include anaemia measurement method in Ethiopia and the cut off as well used in the study for lactating mothers.
5. Still results sections needs improvement and there is mismatch on table no, figure no etc. like figure no. 1, 2, 3, 4 etc has mentioned twice with two different headings.
6. Figures are missing at all in the revised edited version.
7. Authors are proving only spatial hotspot and cold spot of the anaemic lactating women, if I am not wrong. But, important is why these are so, is important question and can be worth of the paper.
8. Maps and some tables, figures are missing at all in the revised paper.
9. Authors can have look on the paper related to their concerned issues. <https://journals.plos.org/plosone/article?id=10.1371/journal.pone.0197171>

Thank you!!!
