# Supplementary material for: Spatiotemporal patterns of anemia among lactating mothers in Ethiopia using data from Ethiopian Demographic and Health Surveys (2005, 2011 and 2016)
Source: PLoS One. 2020 Aug 6;15(8):e0237147. doi: 10.1371/journal.pone.0237147 (PMC7410320; doi:10.1371/journal.pone.0237147)
Supplement: Attachment — Submitted filename: second response to reviewers.docx [file pone.0237147.s004.docx]

**Date May 20/2020**

**To:** PLOS ONE Journal

**Subject**	Submitting Revised Manuscript after Reviewers comment

**Manuscript title:** Spatial Patterns of Anemia among Lactating Mothers in Ethiopia: Data from Ethiopian Demographic and Health Surveys (2005, 2011 and 2016)

**Manuscript ID:** PONE-D-19-30322R1

**Dear editor/reviewers**

We would like to thank you for your constructive comments on our manuscript. We really appreciate your continuous effort and academic commitment to strengthen our paper. Our point-by-point responses for each comment and questions are described as follow. The details of these changes were shown by track changes feature attached.

**Point by point response for reviewers comment**

**For reviewer # 1**

1. Why the spatial hotspot is in those areas. Authors are proving only spatial hotspot and cold spot of the anemic lactating women, if I am not wrong. But, important is why these are so, is important question and can be worth of the paper.

**Author’s response:** Thank you. We are very pleased to respond to this comment which is really important point that we missed in previous reflection.

Though iron supplementation is an essential service for prevention of anemia among lactating mothers, its coverage is very low in these pastoral regions (Afar and Somali) which might be responsible for a high prevalence and hotspot areas of anemia in these regions as compared to other parts of Ethiopia. In addition, the compliance of lactating mothers to the use of iron supplementation survice in these regions is below the recommended level (43). This might lead to an increased risk of anemia among lactating mothers in Afar and Somali regions compared to other regions. The other possible reason behind the consistent hotspot areas in these regions could be nutritional problems such as lack of diatery diversity, consumption of camel and cow milk which had relatively low iron content and the seasonal variation of food they consume (pastoral community). We have included this justification in the revised document (see line 393-401 of the discussion section).

2. Using data for three points of time to make it comparable also needs logically justification.

**Author’s response:** To date, the risk areas (hot spot) of anemia among lactating mothers in Ethiopia are not identified. Thus, this study aimed to explore the spatial pattern of anemia among lactating mothers in Ethiopia over the last one and half-decades at the national level to point out whether there was either the shift or improvement in anemia risk areas following intervention programs in between the survey periods in Ethiopia. Therefore, detecting the geographic variation of anemia during lactation is important to prioritize and design targeted intervention programs to reduce anemia especially in those areas with consistently higher risk of anemia over time (see line 80-86 section of introduction part).

3. Keep the use of term consistent- like somewhere author used maternal, somewhere lactating and somewhere breastfeeding mothers.

**Author’s response:** Thank you reviewer. We have modified accordingly in the revised manuscript (as you can see the track change feature).

4. Line no 50 are not well connected with line no 49.

**Author’s response:** thank you. It is corrected accordingly

5. Authors need to rewrite the data source and sampling section and data measurement section. As these two are overlapping.

**Author’s response:** Thank you. We have reorganized this section in logical and sequential approach

6. Authors missed to include anemia measurement method in Ethiopia and the cutoff point as well used in the study for lactating mothers.

**Author’s response:** Thank you. We have included the measurement of anemia in the revised manuscript (see line 143 -149 of method section).

7. Still results sections needs improvement and there is mismatch on table no, figure no etc. like figure no. 1, 2, 3, 4 etc has mentioned twice with two different headings.

**Author’s response:** Thanks. Sorry for the inconvenience we made. We have corrected accordingly and included in the revised manuscript.

8. Maps and some tables, figures are missing and some are not visible at all in the revised paper.

**Author’s response:** thanks you reviewer. We have reworked all the maps, figures and tables to make them visible. However, as you have observed in the previous two submissions, when we prepare the multi panel figure using GIMP software the quality and visibility of the figure diminishes. Therefore, in this revision we have prepared the figures separately and labeled like Fig 5(a), Fig 5(b), Fig 5(c) e.t.c.

9. Has the statistical analysis been performed appropriately and rigorously?

**Authors response:** We reanalyzed the data rigorously to make the results more visible and readable using appropriate spatial analytic tool. For this purpose we presented each figure separately ( see the revised manuscript).

10. Have the authors made all data underlying the findings in their manuscript fully available?

**Authors response:** All the data underlying the findings were fully available in the manuscript. We declare that the authors did not have any special access privileges that others would not have. The data are publicly available upon reasonable request of the DHS MEASURE website through [archive@measuredhs.com](mailto:archive@measuredhs.com). after being authorized user. We also included this text in the revised manuscript.

11. Is the manuscript presented in an intelligible fashion and written in Standard English?

**Authors response:** we reedited the whole manuscript by consulting senior English language professionals in our university to improve the grammatical quality of the article and to meet the journal standard.

**For reviewer # 2**

1. Authors still need to expand the discussion section by providing more explanation for the results and relating findings to previous studies.

**Authors’ response:** thank you. We have incorporated your suggestion in the revised manuscript. see line 393-401 of the discussion section and line number _366 -370 of this section.

2. Also, weaknesses and strengths stated in the discussion section should be moved to a seperate section titled "Strengths and weaknesses"

**Authors’ response:** We put it in separate section in the revised manuscript.

**Thank you in advance for your constructive comments!!!**
