# Supplementary material for: Spatiotemporal patterns of anemia among lactating mothers in Ethiopia using data from Ethiopian Demographic and Health Surveys (2005, 2011 and 2016)
Source: PLoS One. 2020 Aug 6;15(8):e0237147. doi: 10.1371/journal.pone.0237147 (PMC7410320; doi:10.1371/journal.pone.0237147)
Supplement: Attachment — Submitted filename: third response to reviewers.docx [file pone.0237147.s006.docx]

**Date Jun 23/2020**

**To:** PLOS ONE Journal

**Subject**	Submitting Revised Manuscript after Reviewers comment

**Manuscript title:** Spatiotemporal Patterns of Anemia among Lactating Mothers in Ethiopia using data from Ethiopian Demographic and Health Surveys (2005, 2011 and 2016)

**Manuscript ID:** PONE-D-19-30322R2

**Dear editor/reviewers**

We would like to thank you for your constructive comments on our manuscript. We really appreciate your continuous effort and academic commitment to strengthen our paper. Our point-by-point responses for each comment and questions are described as follow. The details of these changes were shown by track changes feature attached.

**Point by point response for reviewers comment**

**For reviewer # 1**

1. In the conclusion of abstract section- Therefore, public health intervention activities designed in a targeted approach to impact high-risk populations as well as the geographic regions is vital to narrow anemia disparity in Ethiopia’- needs attention for correction the statement ‘is vital to narrow anemia disparity in Ethiopia’.

**Author’s response:** thank you reviewer. We corrected accordingly**. (See** the abstract section line 41-42)

2. To date, the risk areas (hot spot) of anemia among lactating mothers in Ethiopia are not identified- so what are the other studies related to anemia among lactating mothers would be important to justify the statement (Line 91).

**Author’s response:** thanks reviewer we have provided additional citations regarding studies conducted on determinants of anemia among lactating mothers. (We kindly request to see introduction section; line 81-82)

3. Line No 115-120 is not required in the way it has been written. Authors are advised to look for section on data source from various published articles in journal of high repute.

**Author’s response:** Thank you reviewer. We have modified accordingly in the revised manuscript (as you can see the track change feature).

4. In Line number 125, it would be better to mention the age range for ‘reproductive age women’

**Author’s response:** thank you reviewer we specified it as “reproductive age women (15-49)”

5. Data measurement’ heading is not appropriate.

**Author’s response:** Thank you. Since this section describes about how anemia is measured which is our problem of interest. Therefore we changed “data management” to “outcome variable” (see line 129)

6. In line no 164 as the author has used word ‘location data’. I think those were geo-referencing data. So it’s always good to use appropriate word

**Author’s response:** Thank you. We have corrected accordingly. (see line 124 of method section).

7. Within the methodology section, spatial analysis section is improved and well written than the previous one

**Author’s response:** Thanks reviewer for your critical review.

8. Line no 214-218: “The prevalence of anemia during lactation was intermittently increasing across regions in Ethiopia. Even though it had decreased from 2005 to 2011 almost in all regions, its prevalence increased from 2011 to 2016 in all regions including the two administrative cities. The highest prevalence was observed in the Somali regional state (68%) and the Afar region (47%) in 2016”- The statement should be written very carefully. As line no 214 reflects that since 2005, anemia among lactating mother is on increase. But, that is not the case as the authors are stating in the line no-215/216 that it had decreased between 2005 and 2011.

**Author’s response:** thanks you reviewer. We have reinterpreted this section.(we kindly request to see the track change feature)

9. Don’t use like ‘in the following figure’ or ‘in the above figure/map, tables’ etc, as at many place in the manuscript, it has written like that

**Author’s response:** we totally removed such phrases per your comment (see the revised manuscript).

10. As in table 2, keep number of digit identical after the decimal. As somewhere its two digit, somewhere, its three digit in second column. In column 4, keep three digits after decimal. Keep it identical for all the tables

**Authors response:** thank you reviewer we corrected in the revised manuscript. (See the revised manuscript).

11. Line no 253- Can be written as all three consecutive survey periods. Because, all surveys has different meaning

**Authors’ response:** thanks reviewer it is corrected accordingly.

12. Line no 264- 270 needs to be written in well manner as the- In the above figure, HH (High-High) means high rates of anemia surrounded by similar characteristics; HL (High-Low) means high rates of anemia surrounded by low rates of anemia; LH (Low-High) means low rates of anemia cases surrounded by high rates of anemia cases; and LL (Low-Low) means low rates of anemia cases surrounded by similar characteristics. The red (HH) color indicates hotspot areas of anemia, the dark blue (LL) color indicates cold spot areas of anemia, and the dark yellow (HL ) and yellow (LH) colors indicate the outliers.-. Section needs rewrite as writing HL means…., LL means is not the standard writing style in result sections. This is not the right way to interpret the map. The authors can narrate what is emerging out from the said map, not like HL means High-Low. As the Map 5a, 5b, 5c are for three different time period so it would be better to do compare the regions. The color combination should be part of methodology section.

**Authors’ response:** thank you. We have reinterpreted the results from cluster and outlier analysis (we kindly request to see the track change feture)

13. Authors mentioning hot spot, cold spot etc in figure 4,5 6. Which needs proper interpretation like what are those spots, is there any change during the three survey period as authors have written about in very brief in discussion section

**Authors’ response:** thanks reviewer we improved the interpretation.( see line 211-217 of result section)

14. This implies, that the special attention of policy makers for anemia reduction should be in those high-risk areas of the country’- this statement needs correction/modification as per the study objectives.

**Authors’ response;** thanks reviewer we critically modified this statement**. (**See line 266-274 section of discussion)

15. This spatial heterogeneity of anemia clustering was again observed prominently in Afar and Somali regions. This showed that the spatial clustering of anemia is more or less consistently higher in the Afar region in all EDHS surveys and the Somali region in the latest two surveys (2011 and 2016)’- Validate your findings with other available studies or is this new finding emerged from your study for the first time.

**Authors’ response; thank you reviewer.** As to our knowledge concerned it is new finding**.** Thus, it is a little bit difficult to validate with other studies**.**

16.Policy suggestion or public health measures are missing, authors can think on those lines.

**Authors’ response; thank you reviewer.** We really appreciate the comment. We included the policy implication of the findings in the revised document.(we kindly request to see the line 294-299 section of discussion)

17. Overall, there is improvement in the paper than the previous one, but the scientific rigor in interpreting the results emerging from the map is somewhat missing. Discussion section still needs to strengthen. It would be better to reduce the number of maps wherever it’s possible. The paper still needs English editing as in the data and methodology section flow and consistency was missing as well. Authors can also think about the title of the paper.

**Authors’ response;** thank you reviewer. We improved the interpretation, Discussion and English editing issues over all readability of the article in the revised manuscript as it can be observed from the track changes.

**Thank you in advance for your constructive comments!!!**
